# Predictors of 2 year outcomes of juvenile idiopathic arthritis in a multicenter Canadian cohort: the ReACCh out experience

**DOI:** 10.1186/1546-0096-10-S1-A40

**Published:** 2012-07-13

**Authors:** Natalie J Shiff, Kiem G Oen, Jaime Guzman, Nicole A Johnson, Adam Huber, Shirley Tse, Lori B Tucker, Rae SM Yeung, Ciaran M Duffy

**Affiliations:** 1 Alberta Children's Hospital, Calgary, AB, Canada; 2 BC Childrens Hospital, Vancouver, BC, Canada; 3 Hospital for Sick Children, Toronto, ON, Canada; 4 IWK Health Centre, Halifax, NS, Canada; 5 Montreal Children's Hospital, Montreal, QC, Canada; 6 University of British Columbia, Vancouver, BC, Canada; 7 University of Manitoba, Winnipeg, MB, Canada; 8 University of Saskatchewan, Saskatoon, SK, Canada

## Purpose

The ability to predict which children with Juvenile idiopathic Arthritis (JIA) are likely to have worse outcomes would allow a more targeted aggressive approach to initial therapy. We used data from the Research on Arthritis in Canadian Children Emphasizing Outcomes (ReACCh Out), a 16-centre prospective inception cohort of children with newly diagnosed JIA, to identify predictors of 2-year outcomes present during the first 6 months after enrollment.

## Methods

Data was available on 223 to 291 children depending on the analysis. The 2-year clinical outcomes were remission, a Childhood Health Assessment questionnaire (CHAQ) score of ≥0.75, and the Juvenile Arthritis Quality of Life Questionnaire (JAQQ) score. Remission was defined according to Wallace criteria (without ESR or CRP), and combined patients on and off medication. The candidate predictors listed in Table 1 were selected for their clinical relevance and forced in a stepwise manner into regression models. They underwent natural logarithmic transformation (Ln) if not normally distributed. Logistic regression was used for prediction of remission and CHAQ outcomes, and linear regression for the JAQQ score.

**Table 1:** Resuts of regression models using basline and 6 month variables to predict 24 month remision, CHAQ ≥ 0.75, and JAQQ score.

Outcome at month 24	N	Regression	Candidate predictor	OR or β at month 0 (95% CI)	OR or β at month 6 (95% CI)
Remission	223	Logistic	Ln (number of active joints)*	0.72 (0.55, 0.95)	0.63 (0.47, 0.85)
			RF	0.52 (0.14, 2.00)	0.51 (0.13, 1.99)
			Ln (CHAQ at month 0)*	0.61 (0.33, 1.12)	0.49 (0.25, 0.98)
			Ln (onset to first visit)^§^	0.92 (0.68, 1.25)	0.92 (0.67, 1.26)
			Ln (age of onset)^#^	1.07 (0.76, 1.51)	1.16 (0.82, 1.64)

CMAQ ≥ 0.75	291	Logistic	CHAQ ≥ 0.75 at momth 0	7.47 (2.48, 22.46)	2.17 (0.68, 6.89)
			Ln (number of active joints )*	1.02 (0.72, 7.43)	1.25 (0.87, 1.78)
			RF	2.14 (0.67, 6.66)	2.38 (0.72, 7.92)
			Ln (JAQQ at month 0)	1.56 (0.34, 7.10)	2.38 (0.73, 7.75)
			Interaction JAQQ x CHAQQ	1.04 (0.90, 1.21)	1.22 (0.94, 1.59)

JAQQ	270	Linear	Ln (JAWW at month 0)	0.34 (0.02, 0.78)	0.75 (0.42, 1.07)
			Ln (CHAQ at month 0)*	0.43 (-0.06, 0.92)	-0.12 (-0.64, 0.26)
			RF	-0.05 (-0.60, 0.50)	-0.10 (-0.58, 0.38)
			Ln (age of onset)^#^	0.12 (-0.05, 0.28)	0.00 (-0.15, 0.15)
			Enthesitis^¶^	0.01 (-0.35, 0.38)	-0.04 (-0.41, 0.33)
			Interaction JAQQ x CHAQQ	-0.01 (-0.09, 0.07)	0.15 (0.05, 0.26)

*0.5 was added to variable prior to Ln transformation

^§^ time in months

^#^ age in years

^¶^ by history or physical exam

## Results

Table 1 shows results of the regression models. The only significant independent predictor of remission at both month 0 and 6 was the number of active joints, but R^2^ was low (0.06 and 0.09 at month 0 and month 6 respectively). A CHAQ score ≥0.75 was a strong independent predictor of a high 24-month CHAQ at month 0 but not at month 6 (R^2^ of 0.22 and 0.24 respectively). The JAQQ score at both month 0 and month 6 was the only significant independent predictor of the 24 month JAQQ score (R^2^ of 0.09 and 0.23 respectively). There was a non-statistically significant trend for rheumatoid factor positivity to be associated with lesser chance of remission and CHAQ score ≥0.75 at 24 months.

## Conclusion

As hypothesized, most measures at 6 months were better than the baseline measures at predicting 2 year outcomes. Interestingly, CHAQ score at baseline seemed a better predictor of 2 year outcome than CHAQ score at 6 months. Although only a single variable was a significant independent predictor of the outcomes in each model, all 3 models explained a larger proportion of variation in the outcome when the additional variables were included. These results highlight the relatively limited ability to predict disease course at early stage of disease, and should act as an impetus for further research into relevant clinical and biochemical markers of outcome.

## Disclosure

Natalie J. Shiff: None; Kiem G. Oen: None; Jaime Guzman: None; Nicole A. Johnson: None; Adam Huber: None; Shirley Tse: None; Lori B. Tucker: None; Rae S.M. Yeung: None; Ciaran M. Duffy for the ReACCh Out Investigators: None.

